# Experimental Optimization of Tannic Acid-Crosslinked Hydrogels for Neomycin Delivery in Infected Wounds

**DOI:** 10.3390/polym17060770

**Published:** 2025-03-14

**Authors:** Peerapat Chidchai, Kanokwan Singpanna, Supusson Pengnam, Thapakorn Charoenying, Boonnada Pamornpathomkul, Prasopchai Patrojanasophon, Prin Chaksmithanont, Chaiyakarn Pornpitchanarong

**Affiliations:** 1 Pharmaceutical Development of Green Innovations Group (PDGIG), Faculty of Pharmacy, Silpakorn University, Nakhon Pathom 73000, Thailand; 2 Research and Innovation Center for Advanced Therapy Medicinal Products, Faculty of Pharmacy, Silpakorn University, Nakhon Pathom 73000, Thailand; 3 Department of Industrial Pharmacy, Faculty of Pharmacy, Silpakorn University, Nakhon Pathom 73000, Thailand

**Keywords:** hydrogel, neomycin, polyvinyl alcohol, polyvinylpyrrolidone, tannic acid, wound

## Abstract

Wound infections pose a significant challenge in healthcare settings due to prolonged healing times and the emergence of antibiotic-resistant bacteria. Traditional wound dressings often fail to provide sustained drug release, optimal moisture retention, and effective antibacterial protection, leading to suboptimal therapeutic outcomes. This study aimed to optimize and develop neomycin-integrated hydrogels crosslinked via tannic acid (TA) for the treatment of infectious wounds. The hydrogels were optimized using a central composite experimental design. The amounts of polyvinyl alcohol (PVA, 10–20% *w*/*w*) and polyvinylpyrrolidone (PVP, 5–20% *w*/*w*) were varied and mixed with a fixed concentration of TA (1% *w*/*w*) as a crosslinker. The water content (%), water absorption (%), erosion (%), water vapor transmission rate (WVTR), and the mechanical properties of the hydrogels were evaluated. Neomycin was integrated in the optimized hydrogel, and the antibacterial activity against Staphylococcus aureus was studied using a time-kill analysis method. The optimal hydrogel formula contained PVA and PVP at a ratio of 20:19.89 by weight. The resulting hydrogel possessed good physical and mechanical properties and had a water content of 71.86%, water absorption of 124.96%, minimal erosion of 33.08%, and optimal WVTR of 5567 g/m^2^/24 h. Furthermore, the hydrogel showed desirable elasticity, with a Young’s modulus of 474.81 Pa and a tensile strength that could resist breakage upon application. The neomycin-integrated hydrogels inhibited bacterial growth comparably to the neomycin solution (0.5% *w*/*v*). Therefore, TA was proven to be a promising natural crosslinker and the optimized hydrogel was demonstrated to be a propitious platform for neomycin cutaneous application, and which could be used to treat infected wounds in the future.

## 1. Introduction

Wound infections are a major healthcare concern, often leading to prolonged healing times, increased medical costs, and a higher risk of complications such as chronic inflammation or systemic infections [1]. The skin serves as the body’s primary defense against microbial invasion, but injuries compromise this barrier, allowing opportunistic pathogens such as *Staphylococcus aureus* to infiltrate the wound site. These infections can result in delayed tissue regeneration, excessive inflammation, and, in severe cases, the development of antibiotic-resistant bacterial strains. Effective wound management is crucial to prevent infections and facilitate rapid healing [2]. Various medical materials are employed for wound closure, including medicinal plasters, gauze, and other similar materials. Ideal wound dressings should protect the wound from microbes, maintain moisture, and promote hemostasis to accelerate the healing process [3,4].

Traditional wound dressings, such as gauze, medicinal plasters, and cotton bandages, provide basic protection but lack the ability to maintain an optimal healing environment. Many conventional dressings fail to regulate moisture levels, leading to excessive wound dryness or excessive exudate accumulation, both of which can impede the healing process [5,6]. Furthermore, topical antibiotics such as neomycin, mupirocin, and bacitracin are commonly used to treat wound infections but are often limited by rapid drug diffusion, poor retention at the wound site, and the need for frequent reapplication. This results in suboptimal therapeutic efficacy and increased patient discomfort [7]. Given these limitations, there is a growing need for advanced wound dressings that not only provide physical protection but also enhance drug delivery and wound healing [8,9]. This includes the use of hydrogels, nanomaterials, and phototherapy [10,11].

Hydrogels have emerged as next-generation wound dressings due to their unique properties, including their high water retention, biocompatibility, and their ability to facilitate sustained drug release. These three-dimensional polymeric networks can absorb large amounts of exudate while maintaining a moist wound environment, which is essential for cell migration, tissue regeneration, and reduced scarring. Among the various polymers used in hydrogel fabrication, polyvinyl alcohol (PVA) and polyvinylpyrrolidone (PVP) are particularly promising due to their excellent biocompatibility, mechanical strength, and tunable swelling properties [12]. A combination of these polymers allows for enhanced mechanical stability while ensuring a suitable environment for wound healing. Furthermore, tannic acid (TA), a naturally derived polyphenol, is used as a crosslinking agent due to its antibacterial, antioxidant, and biodegradable properties [13,14]. TA is a widely used safe crosslinking agent because of its functional groups, pyrogallol and catechol, which offer several bonding sites for various interactions such as hydrogen bonding, ionic bonding, coordination bonding, and hydrophobic interactions [15,16,17].

To systematically optimize the hydrogel formulation, a Design of Experiments (DOE) approach was employed. DOE is a powerful statistical technique that enables simultaneous assessment of multiple formulation variables while minimizing the number of experimental trials [18]. This method provides a structured and efficient way to explore interactions between factors that influence hydrogel performance, as opposed to traditional one-factor-at-a-time experimentation, which is time-consuming and often overlooks complex variable interactions [19,20]. In this study, a central composite experimental design was used to investigate the effects of varying PVA (10–20% *w*/*w*) and PVP (5–20% *w*/*w*) concentrations on key hydrogel properties, including mechanical strength, water absorption, and degradation resistance. The CCD approach was selected due to its ability to model curvature effects and optimize formulation parameters by considering both individual and interactive factor influences. The statistical model utilized for data analysis was a quadratic regression model, which is well suited for capturing non-linear relationships between independent and dependent variables. This model accounts for both linear and interaction effects, providing a more comprehensive understanding of the factors which influence hydrogel performance. The model was validated through analysis of variance (ANOVA), ensuring statistical significance and predictive accuracy.

This study aimed to develop and optimize TA-crosslinked PVA/PVP hydrogels as a controlled drug delivery system for neomycin-loaded wound dressings. Using DOE, the effects of polymer concentration on hydrogel performance, ensuring a scientifically guided optimization process, were systematically evaluated. The optimized hydrogel formulation was further assessed for its mechanical properties, swelling behavior, erosion resistance, neomycin release kinetics, and antibacterial activity against *S. aureus*. By integrating TA as a multifunctional crosslinker, this study contributes to the advancement of biocompatible hydrogel-based wound dressings, offering a stable, antimicrobial, and effective platform for infected wound treatment. This research presents a significant advancement by offering a highly customizable, bioactive hydrogel dressing with controlled drug delivery, paving the way for an effective, biocompatible, and eco-friendly alternative for treating infected wounds.

## 2. Materials and Methods

### 2.1. Materials

PVA (fully hydrolyzed, M.W. ~60 kDa), PVP (average M.W. ~1300 kDa by LS), TA, neomycin trisulfate salt hydrate (Neo), potassium chloride (KCl, M.W. 74.55 g/mol), sodium chloride (NaCl, M.W. 58.44), sodium phosphate dibasic (Na_2_HPO_4_, M.W. 141.96 g/mol), and potassium phosphate monobasic (KH_2_PO_4_, M.W. 136.09 g/mol) were purchased from Sigma Aldrich (St. Louis, MO, USA). *Staphylococcus aureus* was purchased from ATCC (Rockville, MD, USA). Tryptic soy broth (TSB) was bought from Millipore (Darmstadt, Germany). All other chemicals and solvents were used as received without purification.

### 2.2. Experimental Design and Optimization of Hydrogels

In order to optimize the properties of the hydrogel, an experimental design was used to determine the PVA and PVP content. DesignExpert^®^ software, version 11 (DX 11.1.2.0 version, Stat-Ease Inc., Minneapolis, MN, USA) was utilized to conduct a central composite experimental design. The independent variables were the concentrations of PVA (X_1_) and PVP (X_2_), ranging from 10 to 20 and 5 to 20% *w*/*w*, respectively. The responses or dependent variables measured were the Young’s modulus (Y_1_), tensile strength (Y_2_), percentage of water content (Y_3_), water absorption (Y_4_), percentage of erosion (Y_5_), and water vapor transmission rate (WVTR) (Y_6_). The concentration of TA remained constant at 1% *w*/*w* throughout the experiment. The software conducted a comprehensive analysis of the impact of various polymer concentrations on the properties and characteristics of the hydrogel. This resulted in the generation of an optimized condition.

### 2.3. Preparation of Hydrogels

The hydrogels were fabricated through the freeze–thawing technique to form crosslinking points among the polymer chains and the crosslinker. First, PVA stock solution (20% *w*/*v*) was prepared by gradually adding the polymer to hot water (80–90 °C) and then stirring until it completely dissolved. PVP stock solution was also prepared in deionized water. To prepare the hydrogels, PVA and PVP were combined at different concentrations according to the generated experimental runs from the DesignExpert software, version 11. Then, TA was gradually introduced into the polymer mixture and stirred until homogeneity was achieved. Thereafter, the hydrogel solution was centrifuged at 5000 rpm for 15 min to remove all air bubbles, then poured into an acrylic mold. The resulting solution was frozen at −20 °C for 18 h and subsequently allowed to thaw at room temperature for 6 h which counted as 1 cycle. This cycle was repeated to 3.86 cycles (the last cycle was 15.46 h at −20 °C and 5.16 h at room temperature) which was the reported optimized freeze–thawing cycle for product containing PVA to obtain the final hydrogel product [21].

### 2.4. Mechanical Properties

The mechanical strength of the hydrogel was assessed by a TA.XT Plus texture analyzer (Stable Micro Systems, Godalming, UK), using the tensile mode with a 5-kg load cell. Hydrogel samples were cut into the dimension of 10 × 30 mm and subjected to testing using tensile grips at a test speed of 5.0 mm/s until the point of fracture was reached. Five measurements were taken for each sample. The tensile strength at the maximum force upon breakage and Young’s modulus were measured and the results were reported as mean values along with their corresponding standard deviations (S.Ds.).

### 2.5. Water Content

To assess the water content of the hydrogels, each hydrogel was weighed and then dried in a hot air oven set at 60 ± 2 °C until it reached a constant weight. The weight of the hydrogel before (W_i_) and after (W_d_) drying was measured and calculated to determine the water content using Equation (1).

(1) $$\mathrm{Water}\;\mathrm{content}\;\left(\%\right)=\frac{W_{i}-W_{d}}{W_{i}}\times 100$$

### 2.6. Water Absorption

The water absorption test was carried out in order to examine the hydrogel’s potential for water or exudate absorption. The hydrogels were first weighed (W_i_) and then immersed in deionized water at a controlled temperature of 37 ± 2 °C for 24 h. After that, the hydrogels were re-weighed (W_s_) to determine the weight difference between the dry and soaked samples. This weight difference indicated the amount of water absorbed by the hydrogel. The percentage of water absorption was calculated using Equation (2).

(2) $$\mathrm{Water}\;\mathrm{absorption}\;\left(\%\right)=\frac{W_{s}-W_{i}}{W_{i}}\times 100$$

### 2.7. Erosion

The percentage of erosion determines the hydrogel’s degree of crosslinking. The initial step of the erosion assessment involved drying the hydrogel in a hot air oven set at 60 ± 2 °C for 24 h. The dry weight of each hydrogel sample (W_d_) was then recorded. The dried hydrogel samples were allowed to soak in 20 mL of deionized water at a temperature of 37 ± 2 °C for 24 h, causing them to swell. The swollen hydrogel samples were then placed in a hot air oven at 60 ± 2 °C until they reached a constant weight (W_t_). The percentage of erosion was calculated using Equation (3).

(3) $$\mathrm{Erosion}\;(\%)=\frac{W_{d}-W_{t}}{W_{d}}\times 100$$

### 2.8. Water Vapor Transmission Rate

The water vapor transmission rate (WVTR) test was employed to determine the rate at which moisture permeates through the hydrogels over a specified time period and surface area. The hydrogel samples were cut into circular shapes with a diameter of 1.5 cm and a thickness of 0.3 cm. These circular samples were put on top of the opening of a bottle that measured 1.5 cm in diameter and contained 10 mL of deionized water. The weight of the bottle (W_0_) was measured prior to placing in an environment with a temperature of 37 ± 2 °C, 75% RH for a period of 24 h. Then, the container was weighed again (W_g_), which enabled the calculation of the WVTR at 24 h using Equation (4). A represents the area of the opening of the centrifuge tube in m^2^.

(4) $$\mathrm{WVTR}=\frac{W_{0}-W_{g}}{A}$$

### 2.9. Morphology

To investigate the structure of the optimized hydrogel, a scanning electron microscope (SEM; MIRA 3, Tescan, Brno, Czech Republic) was used. The hydrogels were dried via lyophilization. Afetr that, the hydrogels were coated with gold spray and placed onto the SEM’s metal stub for the analysis.

The hydrogel’s pore size was determined using SEM images, and measurement was performed using ImageJ software (version 1.8.0). The porosity of the hydrogel samples was evaluated by analyzing SEM pictures with the ImageJ program. The thresholding process was used to distinguish between pores and solid material by analyzing pixel intensities. Pixels possessing intensities lower than a predetermined threshold were recognized as pores, while pixels possessing intensities higher than the threshold were classified as solid substances. Porosity was determined by dividing the pore area by the entire area of the picture. The porosity was calculated using Equation (5).

(5) $$\%\;\mathrm{Porosity}=\frac{Area\;of\;pore}{\mathrm{Total}\;\mathrm{area}}\times 100$$

### 2.10. Drug Loading

The optimized hydrogel formulation obtained through the experimental design approach was selected to be incorporated with neomycin (Neo). First, Neo powder was integrated into the optimized hydrogel mixture solution at a concentration of 0.5% *w*/*w*, followed by stirring until homogeneity was achieved. The Neo-integrated hydrogel mixture solutions were then subjected to alternating freeze–thaw cycles to produce the final Neo-integrated hydrogel product. After that, a precisely weighed Neo-integrated hydrogel was crushed and placed in 10 mL of PBS pH 7.4 to extract the drug from the hydrogel. Afterwards, a volume of 100 µL from the obtained solution was gathered and transferred to a 96-well plate for Neo analysis. The analysis was performed using a multimode plate reader called VICTOR NIVO^TM^, PerkinElmer, Waltham, MA, USA. The measurement of absorbance was conducted at a wavelength of 305 nm. The Neo concentration was determined by comparing it to the standard curve and it had an R^2^ ≥ 0.999.

### 2.11. Drug Release

The release profile of Neo-integrated hydrogel was evaluated by immersing 5 g of Neo-integrated hydrogel in 15 mL of PBS with a pH of 7.4. The hydrogel was then incubated at a temperature of 37 ± 2 °C with a shaking speed of 100 rpm in a shaking incubator. Afterwards, 100 μL of the release medium was collected at specific time intervals up to 8 h. An equal volume of fresh PBS was then added to the container at each time point to provide a consistent volume. To ascertain the composition of Neo, a 100 µL portion of the obtained sample was introduced into a 96-well plate, and the absorbance at a wavelength of 305 nm was measured using a multimode plate reader. The cumulative release of Neo was plotted against time to illustrate the release profile of Neo. The Neo solution in PBS with equivalent drug content was placed within the dialysis bag and conducted as the control to demonstrate the drug diffusion.

### 2.12. Time-Kill Assay

A time-kill assay was conducted to investigate the effectiveness of Neo-integrated hydrogel in restraining the growth of *Staphylococcus aureus* over a specified duration [22]. First, bacterial suspensions were prepared at a concentration of 1 × 10^4^ CFU/mL in TSB. Subsequently, 1 g of Neo-integrated hydrogel was added into 10 mL of the bacterial suspension and incubated at 37 ± 2 °C for a duration of 24 h. Then, 100 µL of the sample collected from each predetermined time point was transferred to a 96-well plate. The optical density (O.D.) at 600 nm was measured using a VICTOR NIVO^TM^ multimode plate reader, PerkinElmer, Waltham, MA, USA. Plotting the O.D. against time facilitated the determination of the remaining bacteria post-treatment with the test samples. Neo solution (0.5% *w*/*w*) and TSB were used as positive and negative controls, respectively.

### 2.13. Statistical Analysis

The experimental results were expressed as the mean ± S.D. Statistical analyses, including ANOVA followed by Tukey’s post hoc analysis and independent *t*-test, were performed to identify significant differences (*p* < 0.05).

## 3. Results

### 3.1. The Effect of PVA and PVP on Each Response

The central composite experimental design was used to vary the compositions of the hydrogel patch. The formulation included various proportions of PVA (10–20% *w*/*w*) and PVP (5–20% *w*/*w*), with a fixed concentration of TA (1% *w*/*w*). A total of 11 distinct polymer combinations were generated by the software, as listed in Table 1. The Young’s modulus, tensile strength, water content, water absorption, erosion, and WVTR of 11 hydrogels were reported.

The quadratic model was chosen based on statistical analysis and the central composite experimental design, which allows for the evaluation of both linear and interaction effects of independent variables (PVA and PVP) on multiple response variables. This model was found to best fit the experimental data, with *p*-values < 0.05 and high R^2^ values for most responses, indicating a strong correlation between the input factors (PVA and PVP concentrations) and the measured properties of the hydrogel. A quadratic equation is commonly used in such optimization studies because it captures non-linear relationships that might exist between polymer concentration and hydrogel properties. The relationship between hydrogel properties (measured responses) and input variables derived from the quadratic model was generated from the software and is presented in Equation (6).

(6) $$Y_{i}=b_{0}+b_{1}X_{1}+b_{2}X_{2}+b_{12}X_{1}X_{2}+b_{11}X_{1}^{2}+b_{22}X_{2}^{2}$$

where Y_i_ (i = 1 to 6) signifies the measured responses of hydrogel patches, i.e., Young’s modulus (Y_1_), tensile strength (Y_2_), %water content (Y_3_), water absorption (Y_4_), %erosion (Y_5_), and WVTR (Y_6_). The terms X_1_ and X_2_ represent the experimental factors, e.g., the concentrations of PVA and PVP, respectively.

The coefficient (b) in Equation (6) shows how the input variables relate to the responses. These coefficients represent the influence of PVA (b_1_) and PVP (b_2_) concentrations on each response variable. A positive coefficient denotes an augmenting influence, while a negative coefficient indicates a diminishing impact on the output responses [23]. The coefficient of determination (R^2^) is assessed to validate the suitability of the equation. The coefficient values (b) for each response, along with the *p*-value and R^2^, are given in Table 2. Apparently, Young’s modulus (Y_1_) is positively influenced by PVA and PVP, whereas PVA (coefficient value 267.63) exhibited a greater impact than PVP (a coefficient value of 82.19). Similarly, tensile strength (Y_2_) demonstrated a positive correlation with both PVA and PVP, and PVA with a coefficient value of 3.54 had a higher impact compared to PVP with a coefficient value of 0.8367. The lack-of-fit test evaluates the sufficiency of a regression model by comparing the variability of the data explained by the regression equation to the variability of the data unexplained by the model. A substantial lack of fit, usually shown by a *p*-value < 0.05, indicates that the model does not adequately represent the variability in the data, suggesting a poor fit [24,25]. The coefficients can be used to predict the responses upon varying the concentrations of PVA and PVP, allowing the customization of a hydrogel that was suitable for the applications. Thus, the constraints for using these equations to precisely predict the results were the preparation method and that the chemicals should be in an identical grade as conducted herein.

### 3.2. Mechanical Properties of Hydrogels

**Figure 1:**
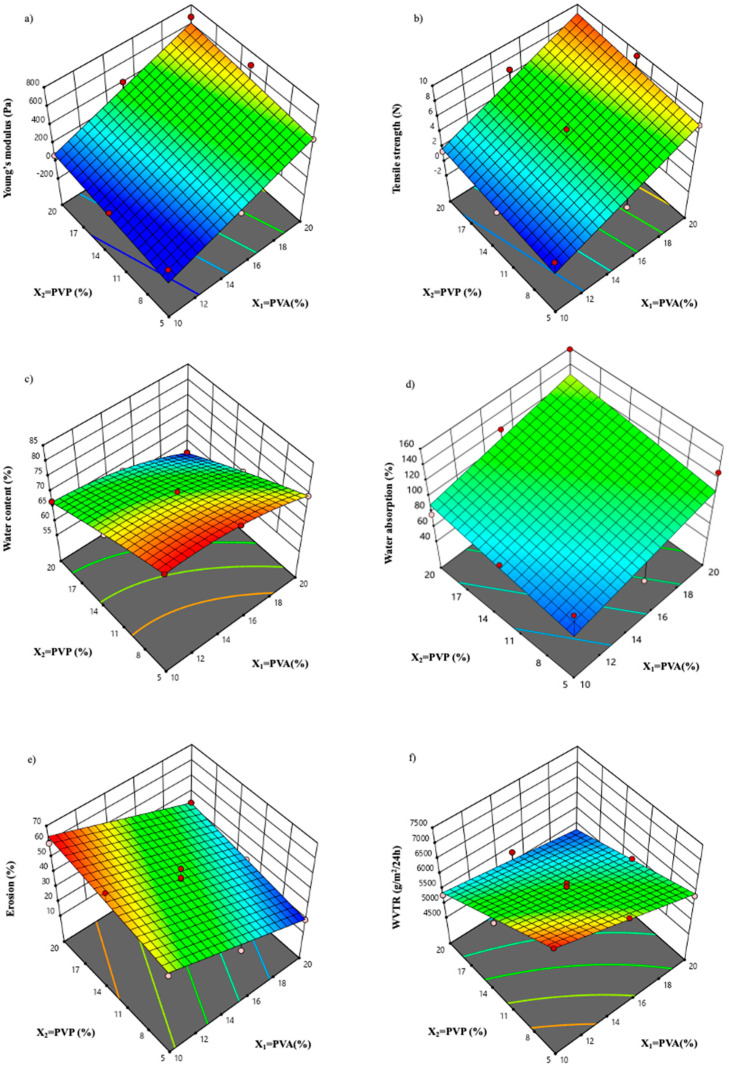
The 3D response surface plots depicting the output responses for (**a**) Young’s modulus, (**b**) tensile strength, (**c**) water content, (**d**) water absorption, (**e**) erosion, and (**f**) WVTR. The blue areas represented the lower values, the red areas represented the higher values.

The Young’s modulus and tensile strength of the hydrogels were evaluated to establish their flexibility and suitability for wound application. The 3D surface plots depicting the effect of PVA and PVP on Young’s modulus and tensile strength are presented in Figure 1a,b, respectively. According to the findings in Table 2, the coefficient value of Young’s modulus (Y_1_) and tensile strength of the hydrogel (Y_2_) indicate a positive influence of PVA and PVP. The concentration of PVA (coefficient value Y_1_, b_1_ = 267.63 and Y_2_, b_1_ = 3.54) had a greater impact than the concentration of PVP (coefficient value Y_1_, b_2_ = 82.19 and Y_2_, b_2_ = 0.8367). Moreover, the results from Figure 1a of the 3D surface plot indicate that increasing the concentrations of PVA and PVP in the hydrogel formulation results in an enhancement of the Young’s modulus and tensile strength. These results suggest that PVA and PVP play a pivotal role in improving the physical properties of hydrogels. Upon mixing PVA and PVP, it is expected that the inter-chain hydrogen bonding between the carbonyl group of PVP and the hydroxyl group of PVA occur [26]. An increase in the concentration of these polymers could lead to a corresponding increase in the crosslinking density, thereby increasing its mechanical strength [27]. However, the lack of fit of the Young’s modulus was significant (*p*-value < 0.05), suggesting that the model did not sufficiently capture the variability caused by unexplained factors in the data. Therefore, utilizing Young’s modulus as a parameter for enhancing the hydrogel was not suitable, as the model could not accurately forecast the hydrogel’s behavior in specific circumstances. On the other hand, the lack of fit of the tensile strength was not significant (*p*-value > 0.05), indicating that the regression model adequately fits the data, suggesting that the model effectively captures the variability in tensile strength. Therefore, the tensile strength parameter appears suitable for use in optimizing the hydrogel formulation. Furthermore, the high R^2^ value of 0.8503 suggests a strong positive relationship between the predictor variables and the response variable, indicating that the concentrations of the polymers in the hydrogel formulation have a significant impact on its tensile strength. Overall, these findings support the use of tensile strength as a criterion for optimizing the hydrogel formulation.

### 3.3. Water Content (%)

Adequate moisture levels play a crucial role in achieving optimal wound healing outcomes. To ensure a proper balance of moisture in the wound bed, the development of hydrogels with appropriate water content is important. As depicted in Table 1, it can be observed that all the hydrogels had high water content of over 50%. The findings in Table 2 show that the coefficient value of the water content of the hydrogel (Y_3_) was negatively influenced by both PVA and PVP. Likewise, the 3D surface response in Figure 1c illustrates a notable trend indicating a reduction in water content with an increase in the concentration of both PVA and PVP. The observed phenomenon can be attributed to the fact that a higher concentration of the polymer translates to a higher crosslink density, consequently leading to a limited capacity for retaining water within the polymer network [28]. The outcomes indicated that the hydrogels comprising PVA at a concentration range of 10–20% *w*/*w* in combination with PVP at a concentration range of 5–20% *w*/*w* are highly promising candidates owing to their high water content. In addition, the lack of fit of the water content was not significant (*p*-value > 0.05), indicating this parameter was appropriate for use in optimizing the hydrogel formulation. The R^2^ value of 0.9981 reinforces the significant correlation between the polymer concentrations in the hydrogel formulation and water content. Overall, these findings support the use of water content as a criterion for optimizing the hydrogel formulation.

### 3.4. Water Absorption

In addition to the water content, the effectiveness of hydrogel dressings in wound healing also depends on their ability to absorb exudate from the wound site. This key attribute can be determined by measuring the percentage of water absorption in hydrogels, as presented in Table 1. The results revealed that the hydrogels exhibited a noteworthy ability to absorb water, ranging from 58.00% to 158.32%. Also, the findings in Table 2 showed that the coefficient value of water absorption (Y_4_) indicates a positive influence from PVA and PVP. Moreover, the 3D surface plot results present in Figure 1d depicted the water absorption characteristics of the hydrogels once the concentrations of PVA and PVP were varied. The findings revealed that an increase in the concentration of both polymers is directly proportional to an increase in water absorption capability. Furthermore, the lack of fit of the water absorption was not significant (*p*-value > 0.05), meaning that this parameter could be used to optimize the formulation of the hydrogel. The R^2^ value of 0.8451 provides further support for the notion that the polymer concentrations in the hydrogel formulation have a significant impact on water absorption capability. In general, these results provide evidence in favor of employing water absorption as a parameter for enhancing the formulation of hydrogels.

### 3.5. Erosion

An erosion test was conducted to evaluate the degradation of hydrogels upon swelling and crosslinking density. Elevated erosion levels indicate increased hydrogel degradation, suggesting that the formulation has a low crosslinking density [29]. According to Table 2, the percentage erosion of hydrogels ranged from 19.62% to 62.53%. As shown in Table 2, the erosion coefficient value (Y_5_) indicates that the concentration of the PVA had a negative impact and the concentration of the PVP presented a positive impact. Furthermore, the 3D surface plot in Figure 1e demonstrates a correlation between the concentrations of PVA and PVP and the resulting percentage of erosion. A notable trend is observed, where an increase in PVA concentration is associated with a decrease in erosion percentage, but an increase in the concentration of PVP is correlated with an increase in erosion. The observed phenomenon could be attributed to the distinct effects of these two polymers on crosslinking density. PVA is commonly linked to the formation of a rigid and highly crosslinked hydrogel structure. Consequently, a reduction in PVA concentration may diminish the density and affect the overall structural integrity of the hydrogel [30], whereas an elevation in PVP concentration would introduce a different crosslinking dynamic or modify existing crosslinks, resulting in a more flexible and less stable structure. This alteration in crosslinking dynamics likely contributes to the observed increase in erosion [31,32]. Furthermore, the erosion’s lack of fit was not significant, suggesting that this parameter could be utilized to optimize the formulation of the hydrogel. The significant correlation between erosion and the concentrations of polymers in the hydrogel formulation is further supported by the R^2^ value of 0.9267. In general, these results provide evidence in favor of employing attrition as a criterion for enhancing the formulation of hydrogel.

### 3.6. Water Vapor Transmission Rate

The water vapor transmission rate (WVTR) test is performed to assess the hydrogel’s capability to permit the permeation of water vapor and gases between the wound and the external environment. An effective wound dressing must possess the ability to regulate moisture levels within the wound, making the evaluation of WVTR for the hydrogels crucial. The dressing component should decelerate moisture loss from the wound site to ensure optimal moisture conditions [33,34]. The water vapor transmissibility of the hydrogels in Table 1 exhibited variations ranging between approximately 4600 and 7300 g/m^2^/24 h. Table 2 demonstrates that the WVTR coefficient value (Y_6_) is adversely affected by the concentration of PVA and PVP. Moreover, the 3D surface plot illustrating the relationship between WVTR and the concentrations of PVA and PVP (Figure 1f) depicts a notable trend that an increase in the concentrations of both PVA and PVP led to a decrease in WVTR. This is attributed to the heightened crosslinking density, resulting in a reduction in the pore size of the hydrogel. Consequently, as the pore size decreases, there is diminished permeation of water and gas exchange through the hydrogels. The strong relationship between WVTR and polymer concentrations in the hydrogel formulation is reinforced by the high R^2^ value of 0.9708 with insignificant lack-of-fit. Overall, these findings support the use of attrition as a criterion to improve the development of hydrogel.

### 3.7. Optimization of Hydrogel

The 3D surface response plots suggest a positive correlation between PVA/PVP and Young’s modulus, tensile strength, and water absorption (%), respectively. On the other hand, the water content (%) and WVTR of the hydrogels decrease as PVA and PVP concentrations increase. Additionally, PVA concentration negatively impacts hydrogel erosion, while PVP concentration has a positive impact. The criteria have been established for hydrogel optimization, as described in Table 3. The criteria utilized to optimize the hydrogel were tensile strength, water content, water absorption, attrition, and WVTR, as determined by the lack of fit. Nevertheless, Young’s modulus was not incorporated into the hydrogel optimization criteria. According to the ideal and desired characteristics of the hydrogels, the optimization criteria were used to maximize tensile strength and %water absorption while minimizing the %erosion. A high tensile strength can provide protection, support, and an optimal environment for cells and tissues to facilitate wound dressing, as well as hydrogel applicability [22,35]. High water absorption was expected to allow more exudate absorption capability, while minimizing erosion was expected to preserve its structural integrity [29]. Furthermore, the in range criteria were chosen for the water content and WVTR, as the values in the range obtained were considerable. The prescribed standards for water content and WVTR are in line with the desired characteristics for wound dressings, while the water content creating a moist environment to facilitate tissue regeneration and preventing the formation of scabs or dry crusts [36,37]. Additionally, WVTR is consistent with the range observed in commercially available wound dressings [38]. In summary, these criteria encompass a thorough strategy for maximizing the effectiveness of hydrogel formulations in promoting wound healing.

According to the software, the PVA and PVP concentrations of the optimized hydrogel were 20.00 and 19.89% *w*/*w*, respectively. This formulation displayed a desirable value of 0.879, signifying a combination of acceptability and excellence. The desirability metric served as a valuable tool for evaluating the multi-response optimization value, with the acceptable and excellent range set between 0.8 and 1. This range indicates that the formulation possesses high-quality characteristics for wound dressing [39]. Furthermore, the Young’s modulus, tensile strength, water content, water absorption, erosion, and WVTR were calculated and predicted from the software as presented in Table 3.

After the optimized condition was obtained, the hydrogels were prepared accordingly, and characterized comprehensively to demonstrate the actual characteristics of the optimized hydrogel compared to the predicted values, as presented in Table 4. While the numerical difference between the predicted solution and the experimented border may seem minor, the optimization process considers multiple responses simultaneously to determine the best overall formulation. The desirability function in DOE methodology balances between different properties. The results indicated no significant difference between predicted and actual values (*p* > 0.05). Furthermore, the optimized hydrogel formulation demonstrated favorable properties, as evidenced by the water content of 71.86%, water absorption of 124.96%, erosion of 33.08%, and WVTR of 5567 g/m^2^/24 h. The results indicate that the optimized hydrogels have the ability to create and sustain a moist environment that promotes tissue regeneration, preventing the formation of scabs or dry crusts [36]. PVA-based hydrogels generally exhibit stable mechanical properties over extended storage when maintained under appropriate conditions, such as controlled temperature and humidity. The presence of tannic acid as a natural crosslinker may further contribute to stability by enhancing intermolecular interactions and minimizing hydrogel degradation. Additionally, the high water absorption capacity of these hydrogels suggests that they can effectively absorb a large amount of exudate. In addition, the optimized hydrogel exhibited excellent flexibility and strength, resistance to degradation, and appropriate water vapor permeability. These findings indicate that the optimized formulation is suitable to be applied to wounds. Moreover, the results supported the application of TA as a natural crosslinker due to its polyphenolic structure, which grant it the ability to form strong hydrogen bonds that enhance the mechanical properties of the hydrogel. Using a naturally derived crosslinker has several advantages, in light of its biocompatibility and biodegradability; however, TA also has intrinsic antibacterial and antioxidant effects which could benefit the wound healing process. Moreover, TA, as a naturally derived crosslinker, can interact with a variety of polymers (both natural and synthetic) through different bonding mechanisms (e.g., hydrogen bonding, hydrophobic interaction), allowing for the tunability of hydrogel properties. This versatility facilitates the design of hydrogels with specific mechanical, chemical, and biological characteristics tailored to specific applications.

**Figure 2:**
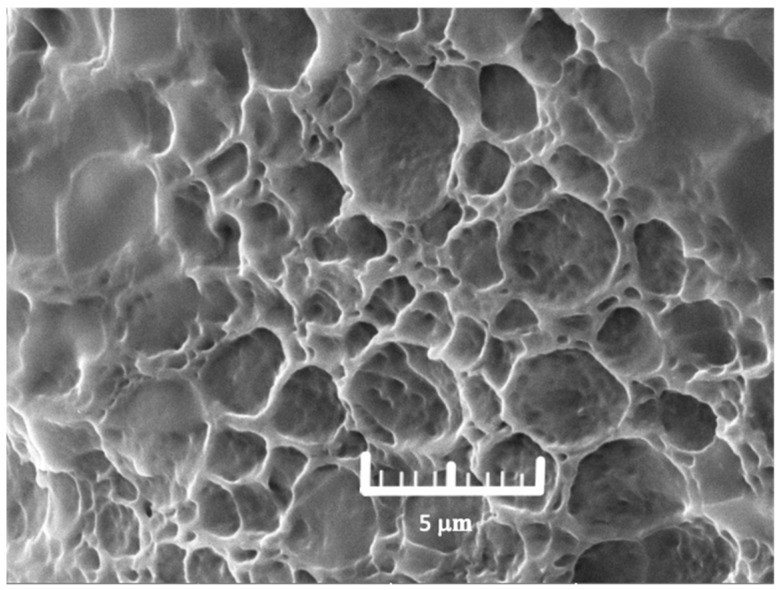
The SEM image of the optimized hydrogel at 1000× magnification.

The SEM micrograph of the optimized hydrogel is depicted in Figure 2. The morphology of the hydrogel patch presented as a compact, porous structure due to the crosslinking of the polymer chains. The pore size of the optimized hydrogel was 3.40 ± 0.28 μm (n = 3), and the % porosity was 59.05 ± 3.57% (n = 3). The relatively low standard deviation in pore size indicates a uniform distribution of pores throughout the hydrogel matrix, which is crucial for maintaining consistent mechanical properties and fluid absorption capabilities. A uniform pore size can facilitate homogeneous water uptake and controlled release, which is particularly beneficial for wound healing applications. Therefore, the presented structure may improve the water absorption capability of the hydrogel as well as allowing water vapor and gas transfer, making it suitable for wound dressing.

### 3.8. Drug Content

The Neo-integrated hydrogel was created by adding Neo to the optimized hydrogel solution at a concentration of 0.5% *w*/*w*, following the formulation of the commercial Neo cream. The drug analysis in the hydrogel showed that the Neo amount was 100.82 ± 2.39% of the expected amount, meeting the acceptable range set by the United States Pharmacopeia 2023. The Neo content falling within the acceptable range suggested that there was no significant drug loss during the hydrogel preparation. The findings confirm the successful creation of a Neo-integrated hydrogel with a dependable and uniform drug content that complies with established pharmaceutical criteria.

### 3.9. Drug Release

**Figure 3:**
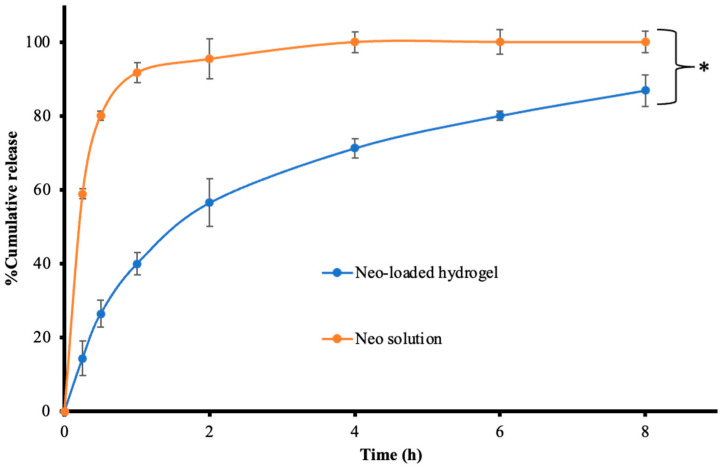
The release profile of Neo-loaded hydrogel and Neo solution (* Significant difference, *p* < 0.05).

The release profile of the Neo-integrated hydrogel (Figure 3) demonstrated a sustained release pattern, with approximately 87% of the drug being released over a 8 h period, whereas the Neo solution exhibited nearly 100% drug release within an hour. The swift release observed in the initial stages was ascribed to the hydrophilic properties of Neo, which allowed it to easily diffuse through the dialysis membrane [40]. The results showed that the Neo-integrated hydrogel had the ability to sustain drug release due to its crosslinked polymeric structure [41]. The Neo-integrated hydrogel exhibited significant swelling in the release medium, leading to a burst release of Neo within 2 h. Subsequently, the hydrogel demonstrated prolonged release of Neo. Additionally, the release profiles of Neo in the medium were modeled using zero-order, first-order, Higuchi, and Korsmeyer–Peppas models, with the model showing the highest linear regression (R^2^) and the highest model selection criteria (MSC) considered the best fit. Also, the release rate constants which allow the prediction of the drug release were calculated and presented in Table 5. The findings illustrated that the release of Neo through the dialysis bag conformed to the first-order model with R^2^ value 0.9942 and MSC 3.5960, emphasizing the impact of concentration gradient on membrane release, aligning with the model’s definition. Conversely, the release of Neo from the hydrogel demonstrated the best fit with Krosmeyer–Peppas kinetic model with R^2^ value 0.9840 and MSC 3.2643. Moreover, the release profile follows Fickian’s diffusion (n ≤ 0.45). This observation can be attributed to the drug having to traverse a swollen hydrogel matrix, where the rate of swelling and the degree of cross-linking in the hydrogel influenced and retarded the drug release rate. These findings suggest the potential of the polymer to serve as a component in a drug delivery system or as a platform for sustained-release drug delivery.

### 3.10. Time Kill Assay

**Figure 4:**
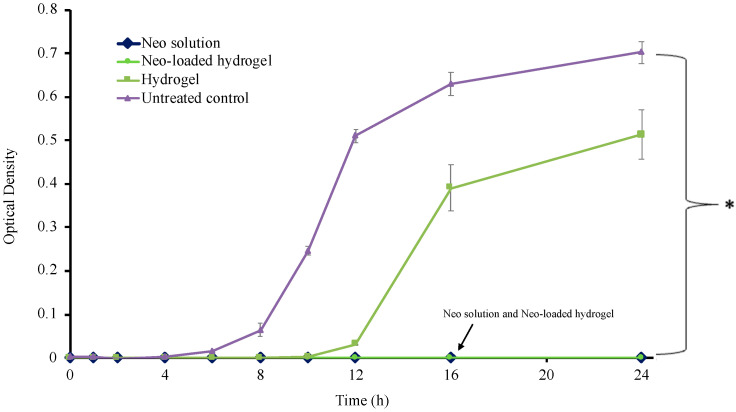
Time-kill release of Neo solution, Neo-loaded hydrogel, blank hydrogel, and untreated control against *S. aureus* (* Significant difference, *p* < 0.05).

The time-kill analysis stands out as an attentive approach for evaluating the bactericidal or fungicidal effects of antimicrobial agents. It provides valuable insights into the dynamic interaction between antimicrobial agents and microbial strains. This method offers crucial information about the effectiveness of the antimicrobial agent over a specific duration of time [42]. Neomycin is categorized as an aminoglycoside antibiotic that has a wide range of effectiveness against both Gram-positive (*S. aureus*) and Gram-negative (*Escherichia coli*, *Klebsiella*, *Proteus*, and *Pseudomonas* spp.) bacteria. It functions by attaching to the 30S ribosomal subunit, which consequently hinders the production of bacterial proteins [43,44]. *S. aureus* is a prevalent bacterial species that frequently causes wound infection. Its presence in wounds can lead to delayed healing and symptoms such as redness, exudate, and abscess formation [45]. In this study, the results obtained from the time-kill assay for *S. aureus* are shown in Figure 4. A higher optical density (O.D.) value indicates microbial growth, while a lower value signifies a bactericidal effect. The findings of this study showed that Neo-integrated hydrogel exhibited a comparable level of antibacterial potency to neomycin solution at all time points. Interestingly, the blank hydrogel also presented some antibacterial effect with reduced O.D. compared to the untreated control. This could be attributed to the crosslinker used. TA was reported to have an antibacterial mechanism by interring the cell wall to interrupt enzymatic activities and inhibiting the synthesis of nucleic acids. Moreover, TA inhibited the bacteria’s attachment to the surfaces, causing bacterial cell death [46]. These results demonstrate that Neo-integrated hydrogel is a practical and effective treatment for infected cutaneous wounds. This therapy has the potential to improve patient outcomes and decrease the likelihood of consequences from bacterial infections. Additional analysis such as morphological analysis using SEM or confocal laser scanning microscopy (CLSM) to observe bacterial structural changes upon interaction with the hydrogel would provide valuable insights into the antibacterial mechanism, including potential membrane damage or biofilm disruption.

## 4. Conclusions

This study successfully optimized a neomycin-integrated hydrogel using a natural crosslinker, TA, for potential use in infectious wound treatment. The application of a central composite experimental design enabled the fine-tuning of hydrogel formulations, specifically adjusting the ratios of PVA and PVP to achieve optimal mechanical and physical properties of favorable water content, and high water absorption. The optimized hydrogel was prepared through the freeze–thawing method, utilizing PVA and PVP concentrations of 20.00% and 19.89% *w*/*w*, respectively, along with 1% *w*/*w* TA. The resulting hydrogels demonstrated excellent water content, absorption, and vapor transmission rates, along with minimal erosion and desirable elasticity, confirming their suitability for wound management applications. The use of TA as a crosslinker not only provided a biocompatible and environmentally friendly alternative but also contributed to the hydrogel’s antibacterial properties, enhancing its effectiveness against common pathogens. This natural polyphenol facilitated the formation of a stable and robust hydrogel matrix, reinforcing the hydrogel’s mechanical strength and prolonging its functionality at the wound site. The integration of neomycin into the optimized hydrogel matrix further validated the platform’s potential for sustained drug release and localized antibacterial action, demonstrating the optimized hydrogel’s considerable promise as a dressing for cutaneous wound infections. A key outcome of this study is the hydrogel’s ability to provide sustained drug release over an extended period. Controlled drug release plays a crucial role in clinical applications by minimizing the need for frequent reapplication, improving patient compliance, and reducing the risk of bacterial resistance resulting from inconsistent antibiotic exposure. The incorporation of tannic acid as a natural crosslinker enhances the structural integrity of the hydrogel while also imparting intrinsic antibacterial and antioxidant properties that may facilitate faster wound healing. In summary, the optimized TA-crosslinked hydrogel presents a promising approach for developing advanced wound dressings that offer controlled drug delivery, enhanced mechanical properties, and reduced patient discomfort. Future studies may focus on in vivo evaluations to further establish the clinical efficacy and safety of this hydrogel system for infected wound applications.

## 5. Limitations and Future Directions

This work focused on establishing extensive characterizations of the hydrogel’s physical, mechanical, and antibacterial properties, which serve as a strong foundation for the potential application of the neomycin-integrated tannic acid-crosslinked hydrogel in wound healing. The hydrogel demonstrated favorable mechanical properties, high water absorption, optimal WVTR, and significant antibacterial efficacy against *Staphylococcus aureus*. These characteristics are essential for wound dressings, as they contribute to maintaining an optimal wound-healing environment by regulating moisture balance, reducing bacterial load, and ensuring mechanical stability for prolonged application.

Despite the advantages demonstrated in vitro, biological environments present additional complexities that cannot be fully replicated under controlled laboratory conditions. Also, further investigation into the stability under storage conditions and during application will enhance the hydrogel’s applicability. The in vivo setting would introduce variables such as immune responses, tissue regeneration dynamics, enzymatic degradation, and blood flow, all of which influence hydrogel performance. Therefore, further validation through in vivo experimentation is required to confirm efficacy and safety.

Subsequent investigations should focus on evaluating hydrogel performance within in vivo wound models to establish its therapeutic potential. Key areas of focus in future research include the hydrogel’s biocompatibility and cytotoxicity, wound closure and tissue regeneration, antibacterial effects on different bacterial strains, and stability.

1. Though biocompatible and biodegradable polymers and crosslinker was used in this study, an examination performed after they have been combined to form the hydrogel would be able to ascertain the formulation’s safety profile.
2. An examination of wound contraction rates, epithelialization, and granulation tissue formation would allow us to determine the hydrogel’s efficacy in promoting tissue repair relative to conventional wound care treatments.
3. Evaluations of the hydrogel’s antimicrobial properties against multiple bacterial species could determine its effectiveness across diverse wound microbiomes. Moreover, an assessment of bacterial clearance in infected wound models could illustrate the antibacterial properties under physiological conditions.
4. Hydrogel’s degradation, mechanical integrity, and drug stability under storage condition should be analyzed.
5. Integration of in vivo findings will provide a comprehensive understanding of hydrogel performance, supporting further optimization and potential clinical translation.

Addressing these factors will not only validate the hydrogel’s therapeutic potential, but will also support future optimization and potential clinical translation, ensuring its effectiveness and safety in real-world medical applications.

## Figures and Tables

**Table 1:** The generated experimental runs from the central composite experimental design for the optimization of PVA/PVP/TA hydrogel.

Run	Concentration (% *w*/*w*)		Young’s Modulus (Pa, Y_1_)	Tensile Strength (N, Y_2_)	Water Content (%, Y_3_)	Water Absorption (%, Y_4_)	Erosion (%, Y_5_)	WVTR (g/m^2^/24 h, Y_6_)
	PVA	PVP						
1	15.00	12.50	149.45	5.60	72.16	69.44	42.56	5888
2	10.00	12.50	40.23	0.43	74.02	82.68	62.53	6210
3	10.00	20.00	70.30	1.46	66.89	77.07	59.23	5299
4	15.00	12.50	97.59	2.77	71.84	86.66	41.76	5878
5	15.00	5.00	149.32	2.50	80.15	58.00	30.26	6685
6	10.00	5.00	80.62	1.81	81.05	95.24	48.05	7370
7	20.00	5.00	444.23	7.20	74.45	127.92	19.12	5855
8	20.00	12.50	672.80	9.44	65.50	86.04	25.95	5383
9	15.00	20.00	417.1	6.78	63.19	140.41	45.62	5348
10	15.00	12.50	151.09	3.61	73.02	90.89	48.45	6004
11	20.00	20.00	679.90	8.29	55.96	158.32	33.11	4616

**Table 2:** Coefficient values (b), *p*-value, and R^2^ for each measured response.

Coefficient	Y_1_	Y_2_	Y_3_	Y_4_	Y_5_	Y_6_
b_0_	268.42	4.54	72.24	97.52	41.51	5866.91
b_1_	267.63	3.54	−4.34	19.55	−15.27	−504.17
b_2_	82.19	0.8367	−8.27	12.44	6.76	−774.50
b_12_	0.00	0.00	−1.08	0.00	0.00	208.00
b_11_	0.00	0.00	−2.32	0.00	0.00	0.00
b_22_	0.00	0.00	−0.4082	0.00	0.00	0.00
*p*-value	0.0014	0.0005	<0.0001	0.0032	<0.0001	<0.0001
R^2^	0.8057	0.8503	0.9981	0.8451	0.9267	0.9708
Lack of fit	0.0482	0.6608	0.8403	0.3678	0.4915	0.1470

**Table 3:** Criteria for hydrogel optimization and the predicted solutions of the optimized hydrogel.

Variables	Criteria	Solutions	Desirability
PVA (% *w*/*w*)	In range	20.00	0.879
PVP (% *w*/*w*)	In range	19.89	
Young’s modulus (Pa)	None	617.078	
Tensile strength (N)	Maximize	4.54	
Water content (%)	In range	72.24	
Water absorption (%)	Maximize	129.33	
Erosion (%)	Minimize	32.90	
WVTR (g/m^2^/24 h)	In range	5867	

**Table 4:** Predicted and actual responses of the optimized hydrogel.

Responses	Young’s Modulus (Pa)	Tensile Strength (N)	Water Content (%)	Water Absorption (%)	Erosion (%)	WVTR (g/m^2^/24 h)
Predicted	617.08 ± 119.08	4.54 ± 1.38	72.24 ± 0.52	129.33 ± 24.74	32.90 ± 4.25	5867± 527
Actual	474.81 ± 15.24	5.82 ± 0.72	71.86 ± 2.33	124.96 ± 3.42	33.08 ± 0.88	5567 ± 351
*p*-value	0.1724	0.2275	0.7964	0.7895	0.9462	0.4623

**Table 5:** Drug release kinetic model represented by the R^2^ of the linear release profile.

Models	Parameters	Neo Solution	Neo-Loaded Hydrogel
Zero order	R^2^	0.0235	0.6029
	MSC	−1.5604	0.3024
	k	17.618	13.441
First order	R^2^	0.9942	0.9434
	MSC	3.596	2.2511
	k	3.325	0.374
Higuchi	R^2^	0.0922	0.9688
	MSC	−1.4609	2.8467
	k	47.84	33.57
Korsmeyer–Peppas	R^2^	0.9643	0.984
	MSC	1.5245	3.2643
	k	83.17	38.14
	n	0.115	0.416

## Data Availability

The original contributions presented in the study are included in the article. Further inquiries can be directed to the corresponding author.
